# Evaluation of *Staphylococcus simulans* inhibition of *Staphylococcus aureus* infection by an *in vivo* murine mastitis model

**DOI:** 10.1128/aem.00678-25

**Published:** 2025-08-04

**Authors:** Benjamin Caddey, Mengyue Li, Jeroen De Buck, Bo Han, Jian Gao, Herman W. Barkema

**Affiliations:** 1 Faculty of Veterinary Medicine, University of Calgary 2129, Calgary, Alberta, Canada; 2 College of Veterinary Medicine, China Agricultural University 34752, Beijing, People’s Republic of China; INRS Armand-Frappier Sante Biotechnologie Research Centre, Laval, Quebec, Canada

**Keywords:** coagulase-negative staphylococci, cattle, bacteriocin, bovine mastitis

## Abstract

*Staphylococcus aureus* is a major bovine mastitis pathogen and can result in chronic intramammary infections that are subject to considerable antimicrobial use. In comparison, non-*aureus* staphylococci (NAS) are common in intramammary infections, but some strains of NAS reduce risk of clinical mastitis and can inhibit *S. aureus* growth *in vitro*. This study aims to determine whether *in vitro* inhibition of *S. aureus* growth by NAS species translates to *in vivo* inhibition within a mouse mastitis model and to characterize NAS mammary tissue colonization. Two *Staphylococcus simulans* strains were selected for *in vivo* experimentation to compare genetically similar strains that can and cannot inhibit *S. aureus in vitro*. Inhibition of *S. aureus* growth *in vivo* was tested by pre-inoculating mouse mammary glands with *S. simulans* 24 hours prior to *S. aureus* inoculation and harvesting mammary gland tissue 24 hours after *S. aureus* superinfection. Mammary glands were processed for bacterial load quantification, cytokine profiling, and histological processing. Pre-inoculation of either *S. simulans* strain resulted in reduced *S. aureus* load in mammary tissue. Superinfection of *S. simulans* and *S. aureus* showed no difference in inflammation severity compared to *S. aureus* alone, although *S. simulans* pre-inoculation significantly increased expression of IL-10. These results demonstrate that protection against *S. aureus* mastitis is possible by prior colonization of mammary tissue by *S. simulans* independent of *in vitro* growth inhibition capacity. This work will foster future research aiming to fully understand the variety of roles NAS strains play in bovine mastitis, aiding in the development toward alternative mastitis prophylaxis.

**IMPORTANCE:**

Bovine mastitis is a leading economic concern for dairy production globally, representing the largest reason for antimicrobial use in dairy cattle. Non-*aureus* staphylococci (NAS) are among the most frequently isolated bacteria from mild, sometimes self-limiting, intramammary infections in cattle and may be associated with a lower risk of infection by major clinical mastitis pathogens such as *Staphylococcus aureus*. This study investigated the inhibition of *S. aureus* mastitis by two NAS strains using an *in vivo* mouse mastitis model. This study demonstrated that when mammary glands are colonized by either one of these NAS strains, the ability of *S. aureus* to establish within the mouse mammary glands is reduced. These results demonstrate the long-term potential for NAS strains to become an alternative prophylactic treatment for bovine mastitis and support efforts to reduce antimicrobial dependencies in food production.

## INTRODUCTION

Bovine mastitis is a leading cause of economic loss and an animal welfare concern in dairy cattle worldwide (1). *Staphylococcus aureus* is one of the most prevalent etiological agents of bovine mastitis and can often result in chronic or serious clinical cases (2). Clinical mastitis cases caused by *S. aureus* are therefore heavily targeted with antimicrobial therapy, raising major concerns of the high antimicrobial use and subsequent increased rates of antimicrobial resistance (2–4). Pressure to decrease use of antimicrobials in livestock has since shifted research focus on mastitis therapy toward alternative treatment strategies (5, 6).

Non-*aureus* staphylococci (NAS) are among the most frequently isolated bacteria from bovine intramammary infections (IMIs) (7). One study of milk samples from more than 5,000 cows has isolated over 20 different NAS species, with a combined IMI prevalence of 26% (8). Their role in the development of bovine mastitis remains unclear; however, IMI caused by NAS is generally subclinical and less severe than those caused by *S. aureus* or other major bovine mastitis pathogens (7). Additionally, in early lactation stages of dairy cattle, some NAS IMI are not associated with any change in milk production and only small decreases in milk quality (9, 10). Some studies even show a slight increase in milk production of NAS-infected cohorts, likely due to an associated reduction in incidence of clinical mastitis, generating further evidence for the potential benefit of certain NAS strains on mastitis prevention (7, 11). One potential mechanism for NAS strains to limit the occurrence of clinical mastitis is through the production of bacteriocins. De Vliegher et al. (12) demonstrated that the bacteriocin production of two *Staphylococcus chromogenes* isolates inhibited *S. aureus* and other important mastitis pathogens, *Streptococcus dysgalactiae* and *Streptococcus uberis*. One study determined that of 441 NAS isolates from the National Cohort of Dairy Farms culture collection of the Canadian Bovine Mastitis and Milk Quality Network (13), 40 isolates representing nine different species were able to produce bacteriocins that inhibited an *S. aureus* clinical mastitis isolate (14). Although the work to establish NAS strains as protective against clinical mastitis is still largely at the *in vitro* phase, there are several studies that demonstrate the protective effect of minor mastitis pathogens (NAS included) against major mastitis pathogens (e.g., *S. aureus*) in murine and bovine mastitis models *in vivo* (15–18). In general, challenge studies demonstrate a reduction in *S. aureus* colonization of mammary glands upon pre-infection with NAS strains, but protective mechanisms are largely unknown.

Some NAS bacteria have a strong potential for protection against severe *S. aureus* mastitis (14). Characterizing the ability of NAS isolates to colonize the mammary glands themselves without causing clinical disease and then evaluating their inhibiting mechanism of pathogens *in vivo* is a clear next step in this research area. Therefore, this study aims to demonstrate the ability of NAS strains with different bacteriocin gene contents to inhibit *S. aureus* infection *in vivo* within a murine mastitis model. Specifically, phylogenetically related NAS strains with different bacteriocin gene clusters will be tested *in vivo* and *in vitro* to investigate potential protective mechanisms against *S. aureus*. The ability of a pair of related NAS strains to colonize mouse mammary tissue and the associated inflammatory consequences will be characterized; meanwhile, inhibitory effects against *S. aureus in vivo* will be compared to *in vitro* models.

## RESULTS

### *In vitro* inhibition of *S. aureus*

**Fig 1:**
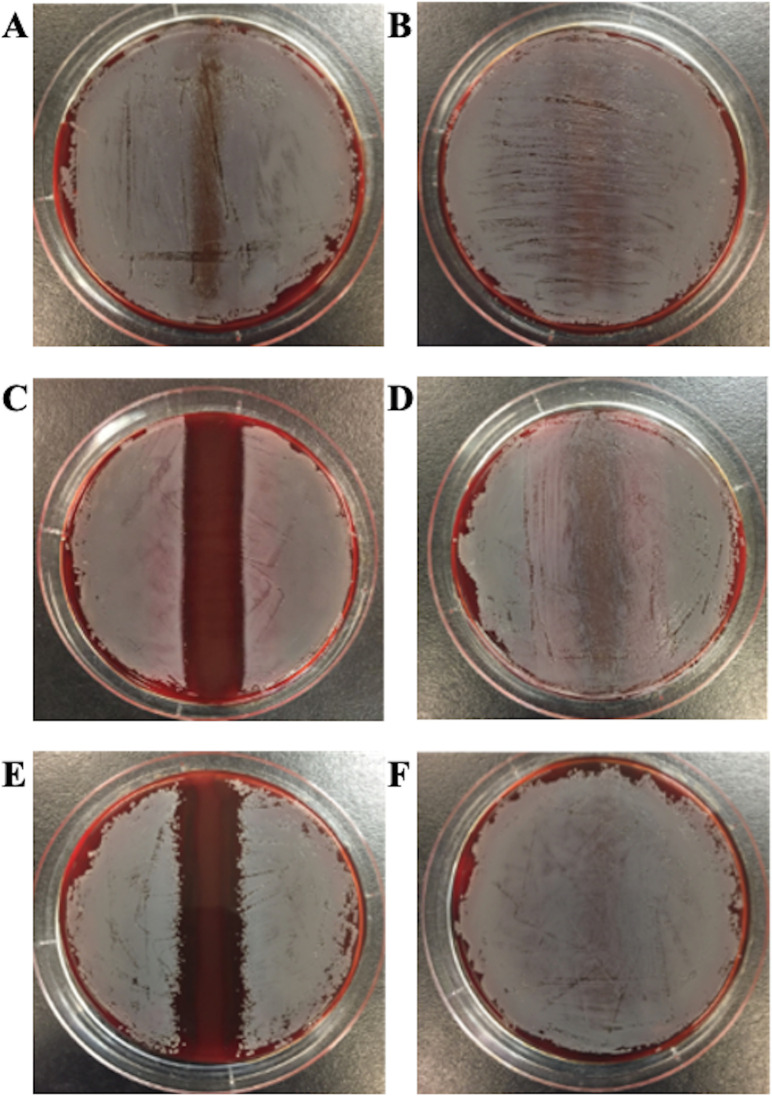
Inhibition of *S. aureus* by NAS strains. *S. aureus* lawn growth on blood agar with inhibiting or non-inhibiting NAS strains cross-streaked on the opposite face of the agar. (**A**) *S. capitis* 4231, (**B**) *S. capitis* 2974, (**C**) *S. simulans* 3061, (**D**) *S. simulans* 1334, (**E**) *S. epidermidis* 1778, (**F**) *S. epidermidis* 2450.

For each NAS species, *Staphylococcus simulans*, *Staphylococcus capitis*, and *Staphylococcus epidermidis*, a previously determined *S. aureus*-inhibiting and non-inhibiting strain were tested for their *in vitro* inhibition ability using a cross-streaking method. All previously determined inhibiting strains of NAS were confirmed to inhibit *S. aureus* growth *in vitro* as presented in Table 1 and Fig. 1. However, *S. capitis* 4231 did not produce a measurable zone of inhibition where it prevented *S. aureus* growth; instead, it only appeared to reduce the growth of *S. aureus*. The width of the zone of inhibition for the other inhibiting strains *S. simulans* 3061 and S. epidermidis 1778 was 16 mm.

**TABLE 1:** *S. aureus* inhibition zones by NAS strains

NAS strain	Inhibition of *S. aureus*	Width of zone of inhibition (mm)^*a*^
*S. capitis 4231*	Yes	0
*S. capitis 2974*	No	–
*S. simulans* 3061	Yes	16
*S. simulans* 1334	No	–
S. epidermidis 1778	Yes	16
*S. epidermidis* 2450	No	–

^*a*^ –, not applicable.

### Determination of inoculum dosage

**Fig 2:**
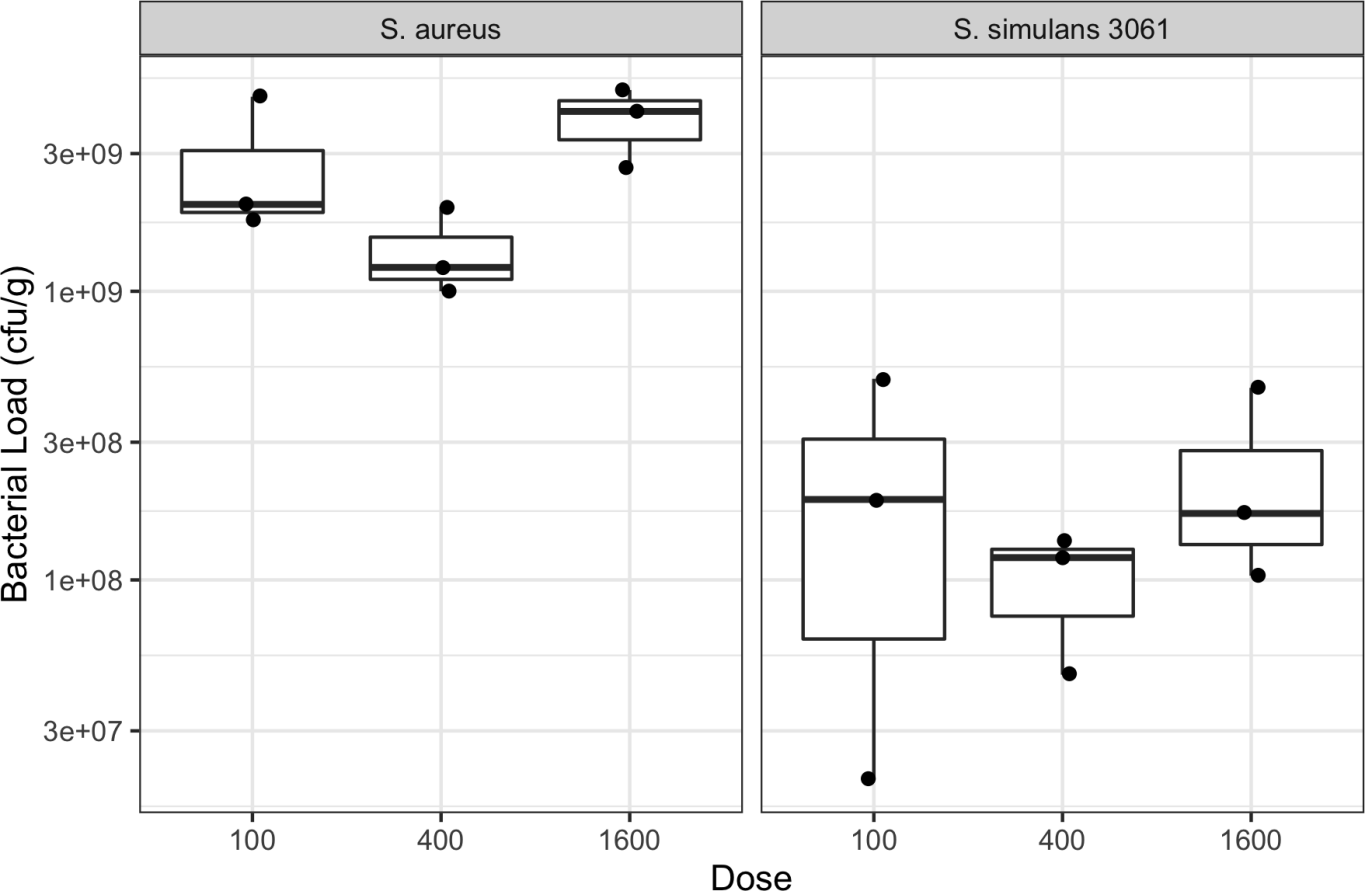
Bacterial load of each *Staphylococcus* sp. recovered from infected murine mammary glands at different inoculum doses. Bacterial load was standardized by CFU per gram of homogenized infected mammary tissue. Boxplots show median, 25th and 75th percentile, 1.5 times interquartile range, with outliers represented as points.

Based on the results from the *in vitro* inhibition study, *S. simulans* 3061 and *S. simulans* 1334 were chosen for *in vivo* experimentation to determine their protective effects against *S. aureus* mastitis. First, the inoculum dosage was optimized for single species inoculation by testing inoculums at three different concentrations, 100, 400, and 1,600 CFU. For both *S. aureus* and *S. simulans* 3061, there were no significant differences across inoculum doses regarding the bacterial load recovered from harvested mammary glands (Fig. 2). In all cases, *S. aureus* inoculated mammary glands had a higher bacterial load recovered compared to *S. simulans* 3061 inoculated mammary glands (Fig. 2).

**Fig 3:**
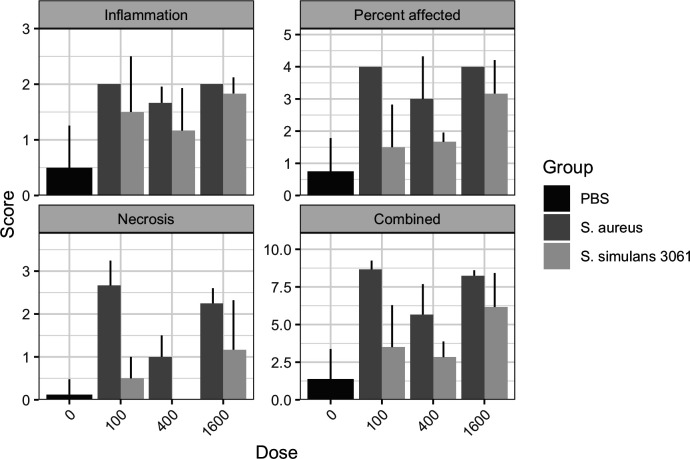
Mammary gland inflammation scores of *Staphylococcus* inoculations at different dosages. PBS was labeled as “0” for inoculum because no bacteria was present in the injected PBS. Inflammation, percent of mammary gland affected, and necrosis scores were all added to get the combined inflammation score. Bars and whiskers represent mean plus standard deviation. Statistical analysis was conducted across groups within the same dosage; no statistically significant differences were identified.

Histological and visual inspections of harvested mammary glands demonstrated that inoculation with either *S. aureus* or *S. simulans* 3061 caused an inflammatory reaction at any inoculum concentration, compared to phosphate buffered saline (PBS)-inoculated controls (Fig. 3). In all measures of inflammation tested for all prepared inoculum doses, *S. aureus* had higher levels of inflammation in harvested mammary tissues (Fig. 3). There were generally no apparent differences in inflammation scores between inoculum doses for either *S. simulans* 3061 or *S. aureus* (Fig. 3). However, for *S. simulans* 3061, the 1,600 CFU dosage scored the highest for percent of mammary tissue affected, necrosis, and combined inflammation compared to the lower CFU dosages (Fig. 3).

### Superinfection of mouse mammary glands by *S. simulans* and *S. aureus*

**Fig 4:**
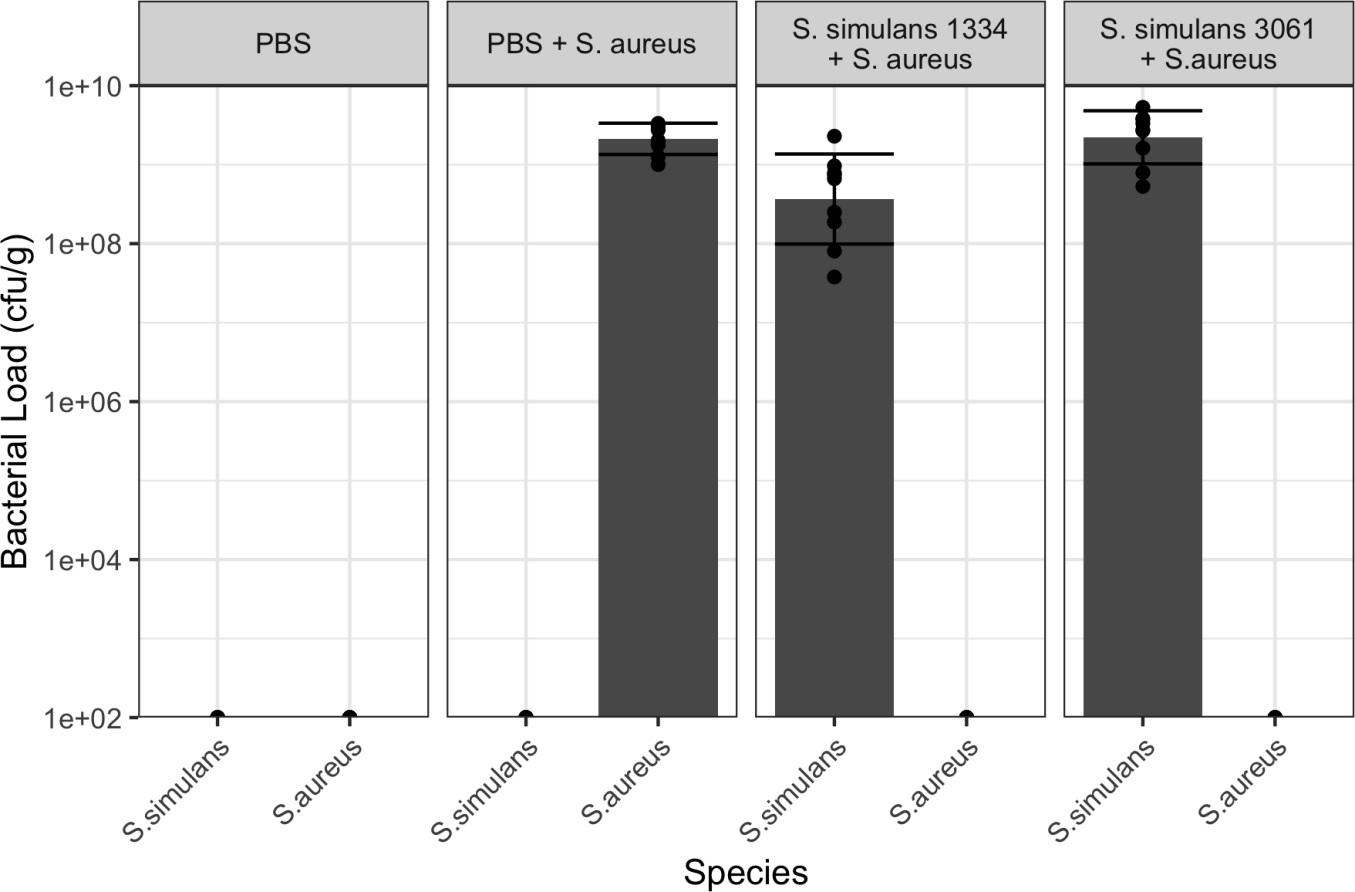
Bacterial load recovered for *Staphylococcus* spp. isolated from superinfected mammary gland infection. Bacterial load was standardized by CFU per gram of homogenized infected mammary tissue. Bars and whiskers represent mean plus/minus standard deviation. No bar or whisker represents no countable CFUs upon serial dilution, while 100 CFUs were calculated as the average limit of detection and set as the baseline for the *y*-axis.

After examining the effects of different bacterial concentrations in the inoculum, dosages of 400 CFU for *S. simulans* 3061 and *S. simulans* 1334 and 100 CFU for *S. aureus* were selected. The bacterial load recovered after 48 hours from superinfections of NAS and *S. aureus* was similar across groups (Fig. 4) and similar to the single species infection models recovered after 24 hours described earlier (Fig. 2). No *S. aureus* were recovered from harvested mammary tissue of mice first inoculated with either 400 CFU of *S. simulans* 3061 or *S. simulans* 1334 prior to *S. aureus* inoculation (Fig. 4).

**Fig 5:**
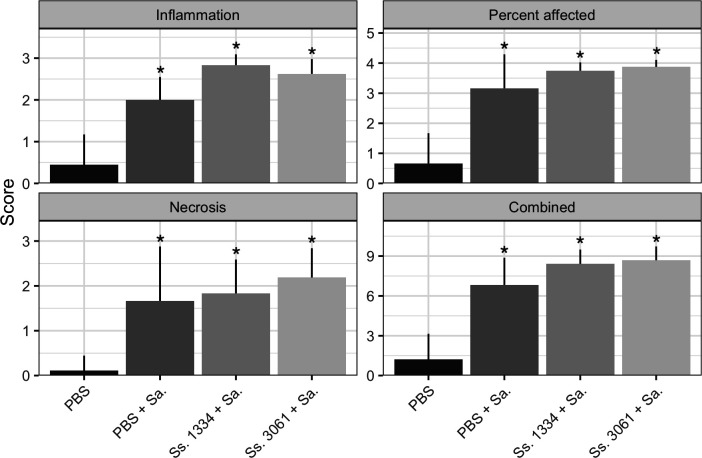
Mammary gland inflammation scores of *S. simulans* and *S. aureus* superinfections. Inflammation, percent of mammary gland affected, and necrosis scores were all added to get the combined inflammation score. Bars and whiskers represent mean plus standard deviation. Asterisks (*) represent significant difference (*P* < 0.05) from bacterial inoculation compared to PBS only. Sa, *S. aureus*. Ss, *S. simulans*.

Inflammation scores of harvested mammary glands are presented in Fig. 5. Average inflammatory scores for PBS-inoculated mammary glands were lower (*P* < 0.05) compared to those with bacterial inoculums, for all categories of inflammation measured. On average, there was no statistical difference between mammary glands inoculated with *S. aureus* and those inoculated with either mixture of NAS with *S. aureus* for all measures of inflammation. Inflammation of mammary glands for all bacterial inoculated groups was generally characterized as moderate to severe; most of the tissue had sparse to moderate necrosis, with moderate inflammatory cell infiltration into the acinar lumen accompanied by interstitial neutrophils and edema.

**Fig 6:**
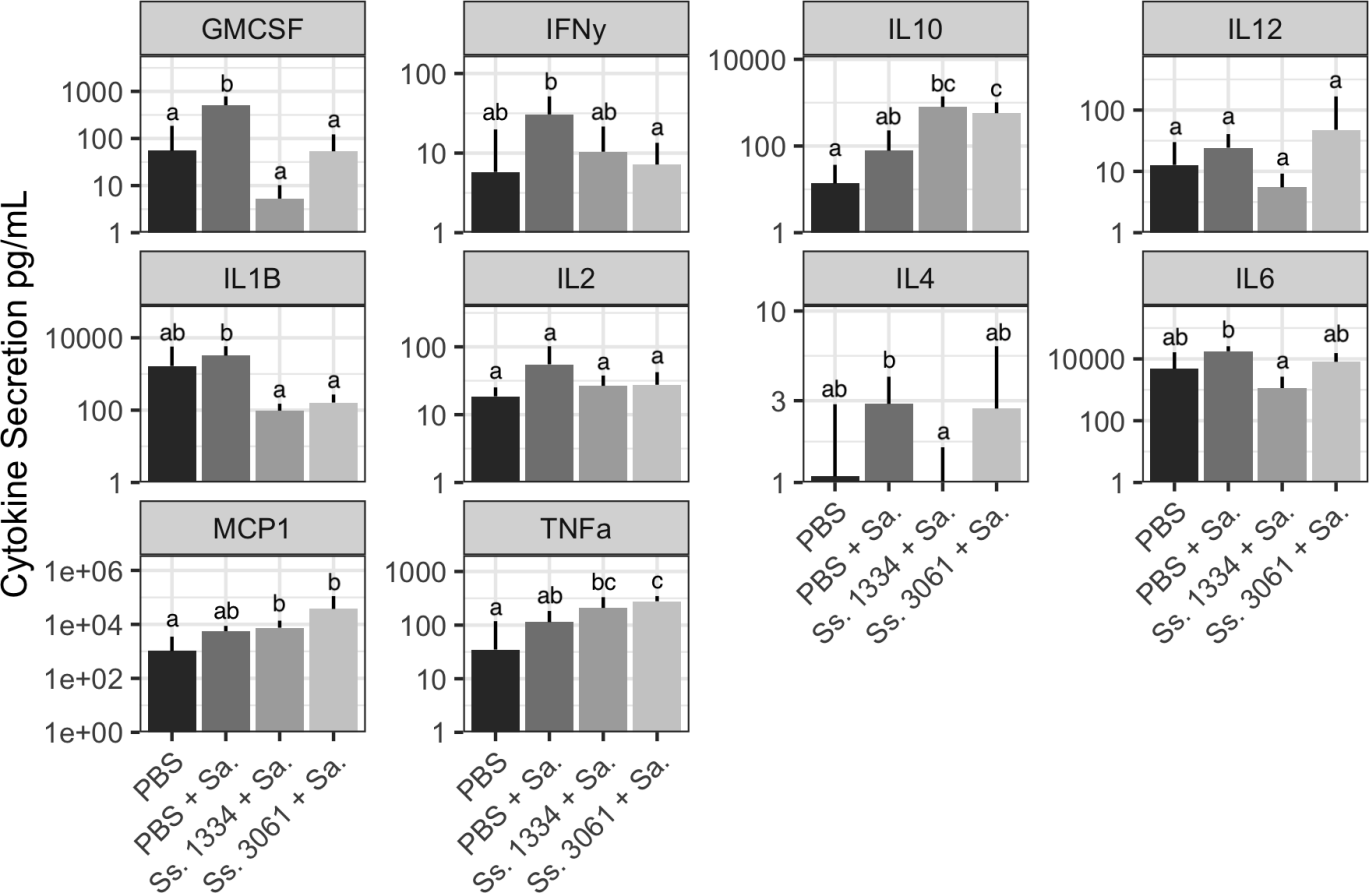
Cytokine quantification from mammary gland homogenate. Cytokine secretion in mammary glands was expressed as pg per mL of mammary gland homogenate. Bars and whiskers represent mean plus standard deviation. Different letters within each cytokine panel represent significant difference (*P* < 0.05). Sa, *S. aureus*. Ss, *S. simulans*.

Inflammatory cytokine expression in the inoculated mammary gland tissue was also measured alongside histological assessment of inflammation and is presented in Fig. 6. Concentrations of granulocyte-macrophage colony-stimulating factor (GM-CSF) and interleukin-1β (IL-1β) were lower in mammary glands inoculated with either *S. simulans* 1334 (*P* = 0.015 and *P* = 0.047, respectively) or *S. simulans* 3061 (*P* = 0.033 and *P* = 0.047, respectively) before *S. aureus* when compared to PBS with *S. aureus* inoculations. Interferon-γ concentration was lower (*P* = 0.033) in mammary tissues inoculated with *S. simulans* 3061 and *S. aureus* compared to PBS with *S. aureus*. Both MCP-1 and TNF-α concentrations were higher in both *S. simulans* 1334 (*P* = 0.029 and *P* = 0.017, respectively) and *S. simulans* 3061 (*P* = 0.017 and *P* = 0.002, respectively) superinfection groups when compared to PBS sham inoculations. IL-10 concentration was higher in mammary glands inoculated with superinfection of *S. simulans* strain 1334 with *S. aureus* when compared to PBS sham inoculations (*P* = 0.009); meanwhile, only the group inoculated with *S. simulans* 3061 expressed a higher concentration of IL-10 compared to PBS with *S. aureus*-inoculated mammary glands (*P* = 0.009).

## DISCUSSION

In this study, we demonstrated a murine mastitis superinfection model for two *S*. *simulans* strains and evaluated their potential to reduce *S. aureus* mastitis. Inoculation of mouse mammary glands of *S. simulans* 3061 or *S. simulans* 1334 24 hours prior to inoculation with *S. aureus* reduced *S. aureus* colonization of the mouse mammary gland tissue. However, inflammation of mammary gland tissues was very similar between the *S. simulans* isolates used in this study and *S. aureus* after the 48hour superinfection model.

There are important limitations of the murine mastitis superinfection model to consider when interpreting the reduction of *S. aureus* growth in superinfections with *S. simulans* presented here. With the current study design, the mechanism of *S. aureus* inhibition in superinfected mammary glands cannot be elucidated. Importantly, it is difficult to distinguish between a specific and direct inhibition of *S. aureus* by *S. simulans in vivo*, or if there are non-specific effects associated with the biology of the pre-infected mammary gland. Alternative possible mechanisms that could alter the ability for *S. aureus* to colonize the mammary glands pre-infected with *S. simulans* include saturation of bacterial load—or bacterial competition for nutrient availability—and the already primed inflammatory state of the mammary gland limiting *S. aureus* establishment. The remaining discussion will focus on broadly characterizing how the different possible mechanisms of *S. aureus* inhibition may have impacted the study outcomes. Further studies must consider the limitations of the study design for murine mastitis superinfection models and should prioritize methodologies that can elucidate the mechanism of pathogen inhibition.

Superinfections starting with either *S. simulans* strain demonstrated reduced *S. aureus* growth *in vivo*, but this was only true for one strain *in vitro*. Regardless of *S. simulans* 3061 inhibiting *S. aureus in vitro* and containing an additional type of bacteriocin gene cluster over *S. simulans* 1334 (14), no difference in their inhibition of *S. aureus* growth *in vivo* was observed. Host-bacteria interactions and inflammatory conditions are likely important factors that could help explain differences between *in vitro* and *in vivo* observations. Nevertheless, because of the usual narrow spectrum of bacteriocins, the lactococcin-like bacteriocin contained by *S. simulans* strains 3061 and 1334 may be a useful tool against *S. aureus* in the future if studied further (19, 20).

The timing of the intervention is critical to reducing *S. aureus* infection, as reported by Brouillette et al. (21), where NAS exoproduct only reduced *S. aureus* colonization of mouse mammary tissue when exoproducts were inoculated before *S. aureus*, which may help explain our observations. Another possible hypothesis outside of bacteriocin production is that some NAS strains, including from *S. simulans*, have demonstrated the ability to reduce the expression of important *S. aureus* virulence factors *in vitro* and may limit *S. aureus* colonization and biofilm formation through quorum sensing systems (22, 23). However, the exact mechanism of *S. aureus* inhibition by *S. simulans* or other NAS *in vivo* is not yet fully understood. Common thought throughout literature is that the bacterial density of NAS and inflammatory state within pre-infected mammary glands are a suggested mechanism of protection against major mastitis pathogens in challenge studies—for example, the pre-infection strain may reach plateau growth in the mammary gland such that growth of competing challenge strains is limited (15, 24, 25). In the present study, inoculating *S. aureus* at 24 hours after *S. simulans*, which were then at concentrations of approximately 2 × 10^8^, likely severely outcompeted the small dose of *S. aureus* subsequently introduced. In general, the longer the interval between protective strain inoculation and pathogen inoculation, the lower the protection against major mastitis pathogens (24, 25). If *S. aureus* growth inhibition is actually through competitive exclusion, additional studies with an altered inoculation strategy are required. Introducing both strains at the same time point, testing different inoculation times for superinfections, and pre-infection with inactivated *S. simulans* strains would be beneficial experiments to elucidate the mechanism of *S. aureus* reduced growth. Overall, a better understanding of the infection kinetics of *S. simulans* and *S. aureus* in mammary glands may provide more insight into the mechanism of protection and persistence of protective effects toward real-world applicability of NAS prophylactic use.

Our study demonstrated that the dose of bacteria within the inoculum has little to no effect on the bacterial load recoverable from harvested mammary glands. Breyne et al. (18) completed a similar mouse mastitis model, using *S. chromogenes* as their NAS species instead of *S. simulans*, and reported a similar lack of dose-dependency. Also, the level of inflammation caused by inoculation of *Staphylococcus* spp. varies considerably in the literature both by species and strain. Comparing NAS isolates against *S. aureus* inflammation, Benites et al. (26) found no significant histopathological differences between the two groups in bovine mammary glands, matching the results from our study. Discordantly, *S. chromogenes* isolates have been demonstrated in different studies to have significantly lower inflammatory results compared to *S. aureus* infection (18, 27); whereas Krishnamoorthy et al. (28) have reported varied results depending on the NAS species tested. Therefore, in future studies, it would be beneficial to fully characterize the severity and duration of inflammation across NAS species and strains to select the most appropriate strain for mastitis therapeutic development.

Inflammatory IL-1β and IL-6 cytokine expression of sham-inoculated mammary glands was similar to *S. aureus*-infected glands. This suggests that there is possibly a baseline inflammatory reaction after clipping mouse nipple ends prior to inoculation. When compared to findings by Breyne et al. (18, 29) who did not clip mouse nipple ends, they found significantly lower cytokine quantities in sham inoculum groups compared to challenged glands. However, there was a reduction in proinflammatory cytokine secretion in the *S. simulans* superinfection groups compared to *S. aureus* and sham inoculum groups (especially IL-1β), suggesting that superinfection with *S. simulans* may help ease inflammatory conditions, emulating previous findings of certain strains of *S. chromogenes* (18). An alternative hypothesis is that NAS-associated inflammation is acute and cytokine expression may dissipate 48 hours post-infection with NAS strains, as compared to other pathogens which have a longer-lasting expression of inflammatory markers (30–32). As stated above, further study of the infection kinetics of *S. simulans* and superinfections with *S. aureus* will provide more clarity of inflammatory processes associated with infection and *S. aureus* inhibition.

Mouse mastitis induced in this study by *S. aureus* was characterized by a broad increase in proinflammatory cytokines, particularly for IFN-γ, IL-1β, IL-6, and GM-CSF to stimulate and recruit macrophages and neutrophils as is expected for *S. aureus* IMIs (33). Mouse mastitis from *S. simulans* and *S. aureus* superinfection was associated with a decrease in GM-CSF but an increase of TNF-α and the anti-inflammatory IL-10. Cytokine production following experimental infection of *S. simulans* has not yet been thoroughly studied, but the observed increased production of IL-10 may point toward these strains as being the causes of persistent IMIs as described in a previous study (34). The general cytokine profile of the superinfection models with either *S. simulans* strains appeared to be less inflammatory compared to mice inoculated with PBS and *S. aureus* only; however, the histological appraisal of mammary gland inflammation indicated a similar inflammatory severity between the two groups. This difference in cytokine expression compared to histological similarity could be explained by the time difference since the start of bacterial inoculation between the two groups. With the superinfection groups, *S. simulans* was incubated within mammary glands for 48 hours before harvesting mammary tissue, while *S. aureus-*only inoculations were incubated at 24 hours. In the dosage experiment of this study, inflammation was scored 24 hours post-inoculation for all groups, and *S. simulans* 3061 had markedly lower levels of inflammation compared to *S. aureus* at all dosages tested. This hypothesis is also supported by the findings by Krishnamoorthy et al. (28), who completed a time-series analysis of inflammation by *Staphylococcus* spp. on mouse mammary glands and found an increased severity of inflammation until 72 hours before proliferation of normal alveolar epithelial cells marked the resolution of intense inflammation. Therefore, in future experiments, it would be advisable to compare the inflammation levels of *S. simulans* from this study with *S. aureus* when both have been inoculated in mammary glands for identical times.

This study provides the necessary foundation for expanding research into the roles of different NAS strains on mammary gland pathology. The effect of the bacteriocin production by each tested *S. simulans* from this study can be further studied by gene knockouts followed by similar *in vitro* and *in vivo* experiments as presented. Additionally, non-specific inhibitory effects of pre-infection by *S. simulans* or other NAS strains should be thoroughly explored to determine the impact of existing inflammation and high bacterial load on *S. aureus* establishment. In general, further *in vivo* experimentation with suspected protective and pathogenic NAS strains combined with phenotypic and genotypic characterization will help elucidate the characteristics and role of NAS species in the mammary gland environment. Protective and less-inflammatory NAS species can also be tested for their inhibition against other persistent and severe mastitis pathogens, such as *Streptococcus agalactiae*, *S. uberis,* and *Escherichia coli*. The mechanisms of protection or pathogenesis by the included NAS species require further investigation for a complete understanding of their potential use on a grander scale for mastitis prevention.

In conclusion, this study demonstrated a murine mastitis superinfection model for *S. simulans* 3061 and *S. simulans* 1334 and evaluated their ability to reduce *S. aureus* growth in mouse mammary glands. Pre-infection with either *S. simulans* strain reduced growth of *S. aureus* inoculated 24 hours later. However, the *in vivo* mechanism of *S. aureus* inhibition is unclear, and additional studies are required to determine the impact of non-specific protective mechanisms on reducing *S. aureus* growth. Furthermore, inflammation caused by both these *S. simulans* strains at 48 hours was also comparable to inflammation following *S. aureus* infection at 24 hours; therefore, any potential protection afforded by these *S. simulans* strains may be outweighed by their deleterious effects. Future investigation is warranted to explore NAS strain differences in their ability and mechanism of mastitis pathogen inhibition to advance the future of alternative treatments against *S. aureus* mastitis.

## MATERIALS AND METHODS

### Bacterial and mouse strains

Bacterial strains used in this trial were originally collected as part of the National Cohort of Dairy Farms, which are stored by the Canadian Bovine Mastitis and Milk Quality Research Network (CBMQRN) (13). In total, six NAS isolates were selected from the collection based on their *in vitro* inhibition of *S. aureus* (14). Inhibiting strains were *S. capitis* 4231 (CBMQRN barcode: 21611308), *S. simulans* 3061 (21308253), and *S. epidermidis* 1778 (20313739). Non-inhibiting strains were *S. capitis* 2974 (41807774), *S. simulans* 1334 (32416176), and *S. epidermidis* 2450 (41605240). The *S. aureus* strain used in this study was isolated from a milk sample of a cow with clinical mastitis (14). All *Staphylococcus* spp. were obtained as frozen glycerol stocks and revived on Columbia blood agar at 37°C for 18–24 hours.

For dose and inhibition experiments, C57BL/6 Elite mice (C57BL/6NCrl; Charles River Laboratories, Wilmington, MA, USA) were used to model bovine mastitis. C57BL/6 Elite mice aged 6–7 weeks were purchased and housed in the Mouse Barrier Unit (MBU) at the University of Calgary. Each batch of purchased mice was reared in the MBU for a week, and then two female mice and one male mouse were placed in the same cage for mating. For the mastitis model, female mice were only inoculated 14 days post-partum.

### *In vitro* screening for NAS inhibition of *S. aureus* growth

To select NAS isolates for inclusion in the mouse mastitis model, inhibitory potential of NAS isolates on *S. aureus* growth was determined using a cross-streaking protocol developed for testing NAS inhibition of *S. aureus* (12, 14). First, a single NAS strain was grown on 5% sheep blood Columbia agar overnight. The isolate was then diluted to a 0.5 McFarland standard in PBS, and 10 µL of this dilution was spread in a line across the central diameter of a fresh blood agar plate and incubated at 37°C for 24 hours. Simultaneously, *S. aureus* was incubated on a separate blood agar plate at 37°C for 24 hours. Following incubation, a sterile inoculation loop was used to release and flip the agar disc of the NAS blood agar so that the clean face of the blood agar strip (opposite the NAS growth) was now accessible for inoculation. From the *S. aureus* culture, a single colony was diluted in PBS to 0.5 McFarland standard, and then an additional 10^−3^ dilution was performed before a 100 µL aliquot was spread evenly over the inverted blood agar and incubated at 37°C for 24 hours. Inhibition by NAS was evaluated by observing the growth of *S. aureus* and measuring the width of the zone of inhibition in the central strip of the plate. A negative control was performed using sterile PBS instead of a NAS isolate.

### Mouse mammary gland inoculation and tissue processing

Inoculation of mice was based on the protocol by Breyne et al. (18). Fourteen days after parturition and 2 hours before mammary gland inoculation, pups and the dam were separated, and the dam body weight was measured. A mixture of ketamine and xylazine was injected intraperitoneally into lactating mice at 50 and 2.5 mg/kg, respectively. Once under anesthesia, buprenorphine was injected to 0.05 mg/kg. Prior to inoculation, mouse nipples and surrounding areas were sanitized with 75% ethanol, and approximately 1 mm of the nipple end was clipped off with scissors. Using a blunt end needle, 50 µL of the inoculum (same inoculum for each pair of mammary glands) was injected into the mammary gland from the opened nipple end. After 24 hours, mammary gland tissue was harvested from the inoculated mice. The mammary glands were visually assessed for clinical signs of inflammation and photographed. A section of the mammary gland (approximately 20 mm long and 2 mm wide) across the length of the gland was excised and stored in 10% formalin for histological analysis. Once removed, the remaining mammary gland tissue was placed individually into pre-weighed gentleMACS M tubes (Miltenyi Biotech, Bergisch Gladbach, Germany), and total weight was recorded to measure mammary gland weight. Mammary tissue was homogenized in 3 mL of PBS using the gentleMACS Octo Dissociator (Miltenyi Biotech, Bergisch Gladbach, Germany). Determination of *Staphylococcus* species recovered from mammary glands was performed by spreading 100 µL of tissue homogenate on CHROMagar (Dalynn Biologicals, Calgary, AB, Canada) and incubated at 37°C overnight. On CHROMagar, *S. aureus* was detectable by its pink colonies and *S. simulans* by their blue colonies. Bacterial load inside the mammary gland was determined by spreading a 10-fold dilution series of 100 µL of tissue homogenate on 5% sheep blood agar and CHROMagar and incubated overnight at 37°C. Colonies were counted and averaged on the two agar plates to determine the bacterial load per gram of mammary tissue.

Histological analysis of the excised portion of the inoculated mammary glands was performed to evaluate the overall inflammation caused by the inoculum. Formalin-fixed mammary glands were embedded in paraffin, and 4 µm sections were stained with hematoxylin and eosin. Inflammation severity was scored based on criteria set by Gogoi-Tiwari et al. (35) and is presented in Table 2.

**TABLE 2:** Histological inflammation scorecard for mouse mammary glands

Measure	Score	Criteria
Inflammation	0	No inflammation: no interstitial and no alveolar inflammatory cell infiltration, no tissue damage
	1	Mild inflammation: small number (<5) of inflammatory cells mainly in the acinar cavity, no tissue damage
	2	Moderate inflammation: 5–20 inflammatory cells, infiltration in acinar lumen, interstitium moderately inflamed with edema
	3	Severe inflammation: >20 neutrophils in all acinar lumens, large portion of necrotic fragments, interstitial neutrophil inflammation and edema
Necrosis	0	No tissue necrosis
	1	Sparse necrosis of acini; less than 10% of gland affected
	2	Moderate necrosis of entire acinus; between 10 and 50% of gland affected
	3	Severe necrosis of entire acinus; >50% of gland affected
Percent of tissue affected	0	No tissue affected
	1	1%–25% affected
	2	26%–50% affected
	3	51%–75% affected
	4	76%–100% affected
Combined Inflammation Score	0–9	Add scores for inflammation, necrosis, and percent of tissue affected

### Determining optimal dose of selected NAS strains and *S. aureus* for inoculation into mouse mammary glands

Based on the results of the *in vitro* inhibition assay described above, the two tested *S. simulans* strains were selected for the mouse mastitis model. These two strains were previously analyzed for bacteriocin gene clusters by Carson et al. (14). The strain, *S. simulans* 1334, contained a lactococcin 972-like gene cluster, whereas *S. simulans* 3061 contained both a lactococcin 972-like and lanthipeptide bacteriocin gene cluster.

To establish a working mastitis model, however, an optimization of the infectious dose was necessary to minimize the *S. aureus* inoculum dose and so that *S. simulans* could both colonize the mammary glands while not causing substantial damage/inflammation itself. The inoculum preparation and infection protocols were adapted from Malachowa et al. (36). Isolates were revived from glycerol stocks on 5% sheep blood agar. Ten colonies were inoculated into 10 mL of brain heart infusion (BHI) broth and incubated shaking at and incubated shaking at and incubated shaking at 37°C for 16–18 hours until a 1:200 dilution was prepared of this growth into 10 mL of fresh BHI, which was incubated until an OD_600_ of 0.75. The bacterial suspension was centrifuged at 3,000 × *g*, at 4°C for 10 min, before washing the pellet with PBS and repeating the centrifugation to isolate the cell pellet. The cell pellet was resuspended in PBS + 25% glycerol till a suspension of OD_600_ 0.3 was achieved and subsequently diluted 1:10 and stored in 50 µL aliquots at −80°C. After a day of storage, CFUs of two aliquots were counted and averaged using a 10-fold dilution series with 5% sheep blood agar to determine the appropriate dilution factors required for inoculum dosage.

Approximately 1 hour before each mouse mammary gland inoculation, 50 µL aliquots of bacterial suspensions were removed from −80°C storage and diluted to the required CFU count. Additionally, 100 µL of bacterial suspension was spread on 5% sheep blood agar to confirm the correct dosage was used for inoculation. Optimal dosage was determined for *S. aureus* (*n* = 3 mice or six mammary glands per inoculum dose) and *S. simulans* 3061 (*n* = 4 mice or eight mammary glands per inoculum dose). Dosage for *S. simulans* 1334 was extrapolated from *S. simulans* 3061 results. Dosages tested were 100, 400, and 1,600 CFU, while PBS was used as a negative control.

### Superinfection trial

To determine the *in vivo* protective effect against *S. aureus* infection by *S. simulans*, a superinfection mouse mastitis model was performed. Mammary gland inoculation protocol was as described above; however, *S. simulans* or PBS was inoculated 24 hours prior to *S. aureus* inoculation. Four experimental groups (*n* = 9 mice; 18 mammary glands inoculated per group) were 400 CFU of *S. simulans* 3061 and after 24 hours, 100 CFU of *S. aureus*; 400 CFU of *S. simulans* 1334 and after 24 hours, 100 CFU of *S. aureus*; 50 µL of PBS and after 24 hours, 100 CFU of *S. aureus*; and 50 µL of PBS. Forty-eight hours after the first inoculation, mammary gland tissue was harvested and processed as described above with one additional step. After preparing the mammary gland tissue homogenate as previously described, 1 mL of homogenate was processed in a series of five centrifugation steps to limit inclusion of cellular debris and lipids from downstream applications: step 1, 307 × *g* for 7 minutes at 4°C; step 2, transfer supernatant to another tube and centrifuge at 5,057 × *g* for 10 minutes at 4°C; step 3, transfer supernatant to another tube and centrifuge at 20,817 × *g* for 10 minutes at 4°C; and repeat steps 2 and 3. The final supernatant was stored at −80°C for cytokine quantification at Eve Technologies Corporation (Calgary, AB, Canada) using their Mouse Focused 10-Plex Discovery Assay.

### Data analyses

Results for technical replicates (pairs of mammary glands for each mouse) were averaged for each mouse, and all subsequent analysis was performed at the mouse level. Statistical analyses were performed using non-parametric Kruskal-Wallis rank sum tests, followed by pairwise Wilcoxon rank sum tests. Multiple hypothesis testing was adjusted using the Bonferroni correction. All analysis and figures were completed using R version 4.2.1 (R Core Team, 2022, R: a language and environment for statistical computing, R Foundation for Statistical Computing, Vienna, Austria).
